# *In Vitro* Cell Proliferation and Mechanical Behaviors Observed in Porous Zirconia Ceramics

**DOI:** 10.3390/ma9040218

**Published:** 2016-03-23

**Authors:** Jing Li, Xiaobei Wang, Yuanhua Lin, Xuliang Deng, Ming Li, Cewen Nan

**Affiliations:** 1 State Key Laboratory of New Ceramics and Fine Processing, School of Materials Science and Engineering, Tsinghua University, Beijing 100084, China; ljing12@mails.tsinghua.edu.cn (J.L.); lim@tsinghua.edu.cn (M.L.); cwnan@mail.tsinghua.edu.cn (C.N.); 2 School & Hospital of Stomatology, Department of Geriatric Dentistry, Peking University, Beijing 100081, China; xiaobei0619@163.com (X.W.); kqdengxuliang@bjmu.edu.cn (X.D.)

**Keywords:** zirconia ceramics, porous, proliferation

## Abstract

Zirconia ceramics with porous structure have been prepared by solid-state reaction using yttria-stabilized zirconia and stearic acid powders. Analysis of its microstructure and phase composition revealed that a pure zirconia phase can be obtained. Our results indicated that its porosity and pore size as well as the mechanical characteristics can be tuned by changing the content of stearic acid powder. The optimal porosity and pore size of zirconia ceramic samples can be effective for the increase of surface roughness, which results in higher cell proliferation values without destroying the mechanical properties.

## 1. Introduction

Due to their excellent mechanical properties, zirconia ceramics have been employed in biomedical applications as early as 1969 [1,2,3,4]. There are three different patterns of ZrO_2_ crystal at ambient pressure, namely, monoclinic, tetragonal, and cubic. Some dopants such as Y_2_O_3_, MgO, or CaO help stabilize the tetragonal phase of zirconia at room temperature, which is beneficial to induce T-M transformation and arrest crack propagation under force, which contributes to the material’s high toughness [2,5]. Compared to conventional alloys and resin materials, tetragonal zirconia ceramics exhibits high strength and toughness, with a flexural strength ranging from 800 to 1000 MPa and a fracture toughness in the 6–10 MPa m^0.5^ range [6]. It also has good chemical stability, with strong resistance to various aggressive chemical agents [7] and great aesthetic value due to its tooth-like color and special translucencies [8].

Furthermore, biocompatibility is highly essential for a biomaterial, which mainly depends on the responses of cells on the materials surface, including cell attachment, cell migration, cell proliferation, and cell differentiation [9,10,11]. All cell activities can fundamentally affect the vital behavior, osseointegration [12,13,14,15]. Reports in wide-ranging literature reveal that surface roughness is needed in biomaterials for osseointegration or adhesion purposes [16,17,18]. Bächle *et al.* [19] investigated the behavior of CAL_72_ osteoblast-like cells cultured on the zirconia ceramics with different surfaces. It was found that, compared with machined ones, cell proliferation values were significantly greater for surface-treated zirconia. Moreover, Yamashita *et al.* [20] found that specimens with rough surfaces had better initial cell responses, compared with the smooth surface specimens.

Several treatments can obtain the needed roughness, but the most commonly employed methods are sandblasting [21,22] and chemical etching [23,24]. In order to increase the surface roughness and area, dental crowns employed in clinic are usually treated by sandblasting, which results in mechanical interlocking between crowns and surrounding restorations [25]. However, the high-speed hard particles generate subsurface cracks while removing pieces of materials to increase roughness. Masses of cracks varying in sizes and directions are likely to produce destructive impairment to the performance of materials [6]. Chemical etching using HF has been proven to be an efficient method to generate roughness on zirconia ceramics, which makes zirconia a good implant with high removal torque values [26]. Whereas, HF is highly toxic to our knowledge and hard to operate with.

Inspired by desirable properties of porous scaffolds with regard to great compatibility, the aim of this study was to produce surface roughness on zirconia ceramics by synthesizing porous ceramics using stearic acid, and to investigate both mechanical properties and cell proliferation behavior as a function of stearic acid content. The null hypothesis of this study was that porous structure would increase the surface roughness of zirconia ceramics, without destroying its mechanical properties.

## 2. Results and Discussion

### 2.1. Phase Structural and Microstructural Analysis

**Figure 1:**
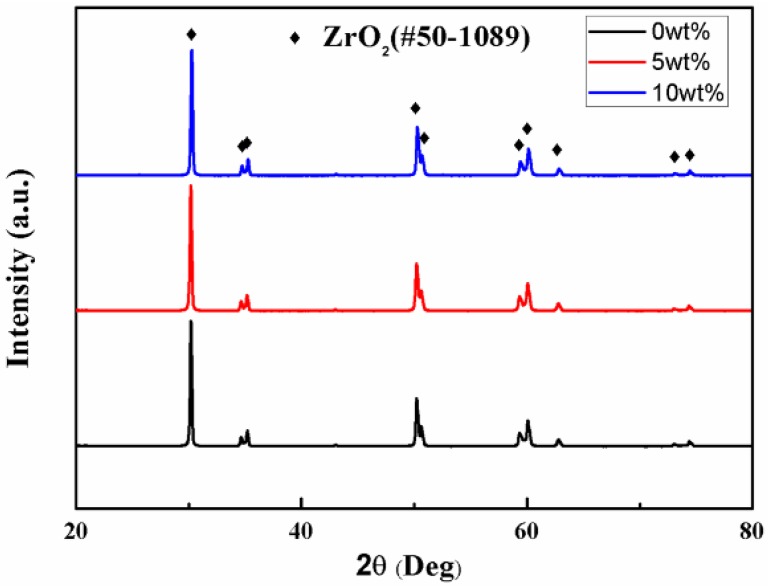
XRD patterns of zirconia ceramics with different stearic acid contents.

The X-ray diffraction (XRD) patterns of zirconia specimens are shown in Figure 1. The characteristic peaks demonstrate the crystalline phase of tetragonal ZrO_2_ indexed with PDF card #50-1089. No secondary phase or processing residue are observed.

**Figure 2:**
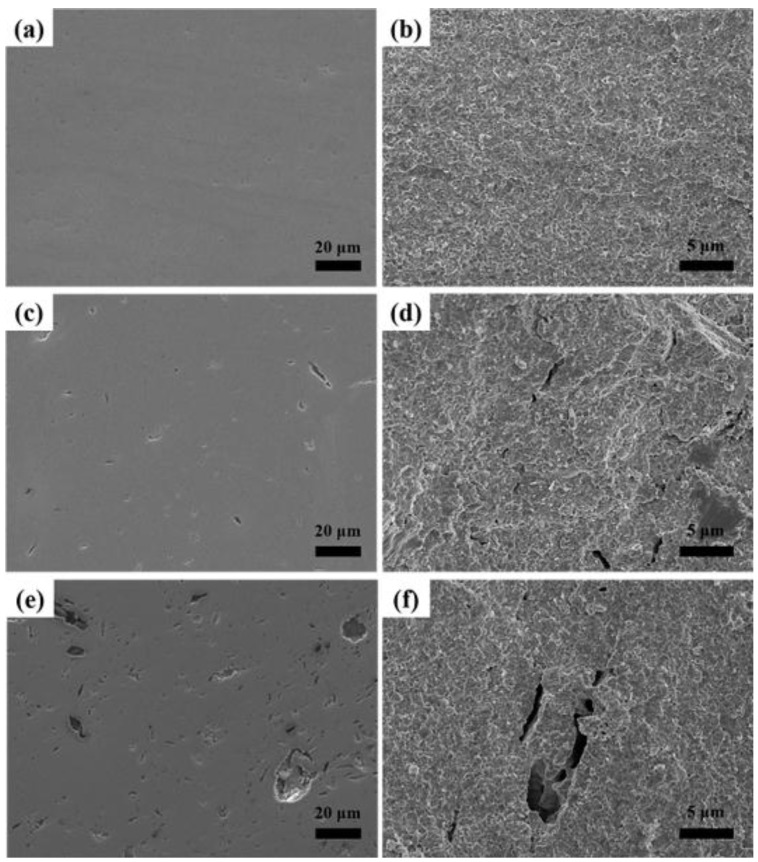
SEM images of polished surfaces and fracture surfaces of zirconia ceramics with different stearic acid contents. (**a**,**c**,**e**) polished surfaces; (**b**,**d**,**f**) fracture surfaces; (**a**,**b**) 0 wt % stearic acid; (**c**,**d**) 5 wt % stearic acid; (**e**,**f**) 10 wt % stearic acid.

SEM images of polished surfaces and fracture surfaces of zirconia ceramics with different stearic acid contents are shown in Figure 2. It is clearly observed that the porosity and the pore size increase with the addition of the stearic acid as pore-forming agent and pore sizes are in the range of 1–20 μm. The shape of most of pores is irregular and becomes partially interconnected when the concentration of the pore-forming agent increases to 10 wt % according to the fracture surfaces in Figure 2. The stearic acid particles turn out to have irregular shapes after ball milling with zirconia grinding balls, leaving pores with similar structures after decomposition during the heating process. At the same time, with increasing amount of pore-forming agent, aggregation deteriorates the irregular shape of the stearic acid particles and introduces interconnected pores with a larger size shown in Figure 2.

**Figure 3:**
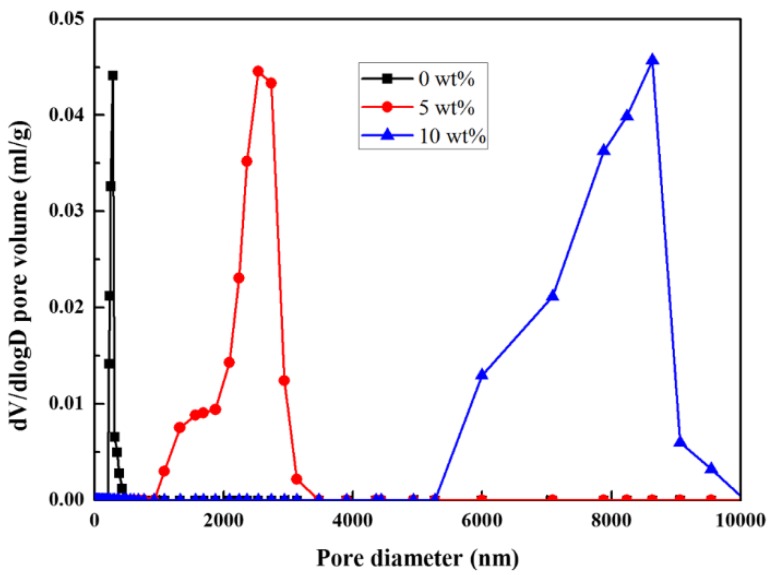
Pore size distribution of porous zirconia ceramics with different stearic acid content.

Figure 3 shows that stearic acid content has significant effects on the mean pore size distribution of the zirconia ceramics. The pore diameter increases with stearic acid content. As seen in the figure, the dense specimen without stearic acid has pore size within nanometer range, whereas the pore diameter of specimen with 5 wt % stearic acid increases to 1–3 μm and that of specimen with 10 wt % stearic acid is in the range of 5–10 μm.

### 2.2. Mechanical Properties

In similar treatments, the linear shrinkage increases slightly with the content of the stearic acid, shown in the Table 1. Compared with zirconia powder, stearic acid has a lower density, resulting in a larger volume with the same mass, which may be responsible for the small increase from 21.6% to 22.1% in the linear shrinkage during sintering.

As the amount of stearic acid increases, the relative density decreases due to the increase of pores by the agent. Based on relative density, the bulk porosity of porous zirconia ceramics is calculated using the following equation:

*p* = (1 − ρ) × 100% (1)

*p* is the bulk porosity and ρ is the relative density). The porosities of specimens are 1.1%, 5.8% and 16% due to different mass fractions of stearic acid, which are 0 wt %, 5 wt %, and 10 wt %, respectively.

Table 1 also shows *R*_a_ (surface roughness, arithmetical mean deviation of the profile) of specimens with different stearic acid content. The surface roughness *R*_a_ of the dense zirconia is 20 nm on average, and that of porous specimens has an average of 50.3 nm and 70.7 nm with increasing content of stearic acid.

**Figure 4:**
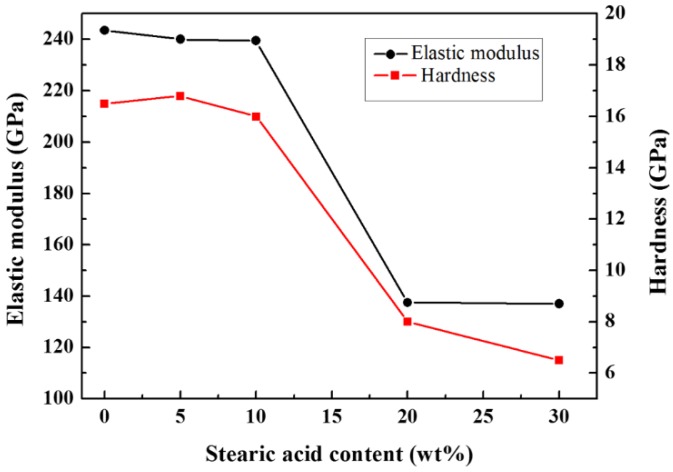
The hardness and elastic modulus of samples with different stearic acid contents.

Figure 4 shows the Vickers hardness and elastic modulus value as a function of stearic acid content. Due to stress concentrations under load, the mechanical properties are impaired by pores. Both the hardness and elastic modulus decrease as the amount of stearic acid increases. Samples with higher porosity exhibit weaker mechanical properties, contributing to a sharp decline in the elastic modulus and hardness in Figure 3. When the stearic acid content exceeds 10 wt %, the hardness value is below 10 GPa and the elastic modulus value is below 150 GPa. In order to ensure sufficient strength against oral mastication, the compromise in mechanical properties sets an upper limit of 10 wt % for the pore-forming agent content and hence experimental groups containing 20 wt % and 30 wt % stearic acid are discarded.

### 2.3. Morphologies and Proliferation of rBMSCs

The porous structure serves as a template for cell contact, migration proliferation, and vascularization, helping cells to attach on the surface and build a three-dimensional spreading-out structure, which promotes cell growth. On the other hand, high porosity and large pore size help the transportation of oxygen and nutrients [27]. The porosity caused by the decomposition of stearic acid increases the surface roughness, which improves the surface wettability and results in a higher cell proliferation values compared with dense zirconia [28].

**Figure 5:**
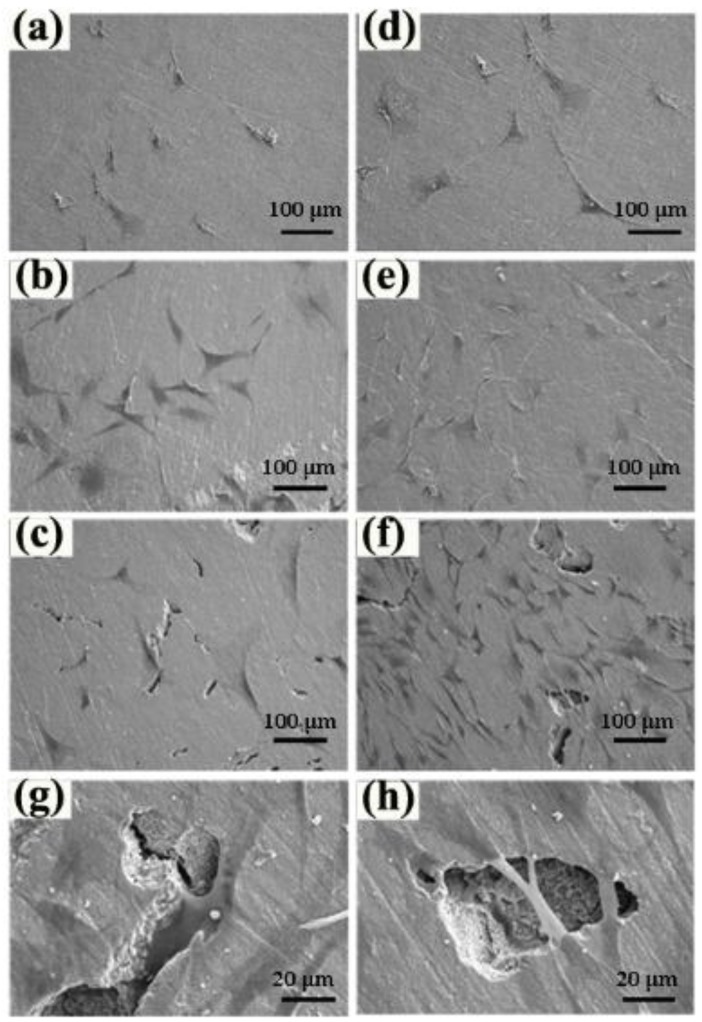
Morphologies of rBMSCs after one and five days culture. (**a**–**c**) the morphologies of rBMSCs after 1 day culture; (**d**–**f**) the morphologies of rBMSCs after five days culture; (**a**,**d**) 0 wt % stearic acid; (**b**,**e**) 5 wt % stearic acid; (**c**,**f**) 10 wt % stearic acid; (**g**,**h**) magnified images of picture.

rBMSCs seeded onto zirconia ceramics show a fibroblast-like morphology without apparent differences in Figure 5. Compared with those observed on the first day, cells grow more vigorously after five days of culturing, demonstrated by more fusiform cells and larger cell spreading areas on the three kinds of specimens. The long-spindle shaped cells are more likely to spread on the specimen with the highest porosity content, indicating that pore structure obviously stimulates the proliferation of rBMSCs *in vitro*. Furthermore, the morphology of pores, to varying degrees, affects the growth of rBMSCs (Figure 5). Cells grown in the tiny spindle cracks lead to mechanical interlocking with specimens, which consolidates their attachment and results in rBMSCs spreading out with overlapping layers on the bottom and across the surface of the shallow disc-shaped pores, thereby promoting cells’ proliferation and spreading out.

**Figure 6:**
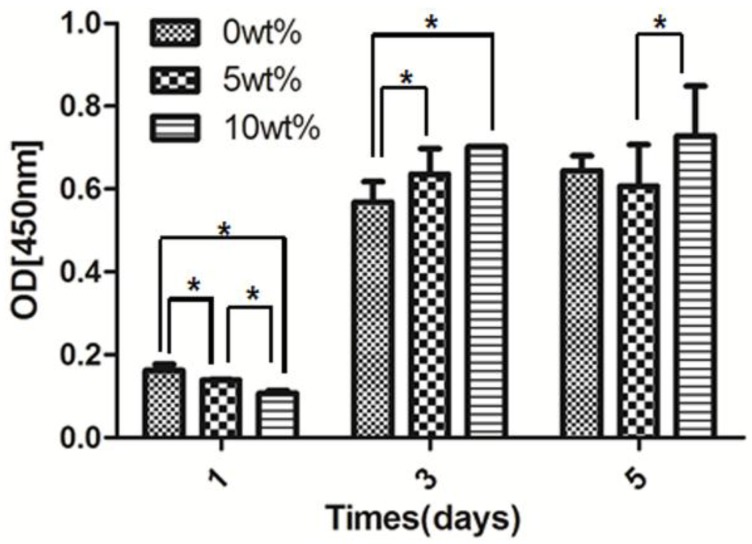
The proliferation of rBMSCs grown on various surfaces by CCK-8 assay after one, three, and five days culture. (* presented *P* < 0.05).

CCK-8 is used to assess the proliferation of rBMSCs on zirconia specimens (Figure 6). Compared with the first day of culture, there is an obvious increase in the OD (optical density) value on day three and five for zirconia ceramics with various pore structures. On specimens containing 10 wt % stearic acid, the number of cells keep increasing from days one to five and stays higher than ones on group with 5 wt % stearic acid by day three and five. Cell number on group with 5 wt % stearic acid increases on day three and decreases on day five, suggesting contact inhibition.

## 3. Materials and Methods

### 3.1. Materials

3Y-TZP (TZ-3YSB-E, Tosoh Co., Tokyo, Japan) with an average particle size of 90 nm and stearic acid (Sinopharm Chemical Reagent Co., Shanghai, China) were used to prepare porous zirconia ceramic. Glutaraldehyde was purchased from Sinopharm Chemical Reagent Co. (Shanghai, China). FBS and rBMSCs were manufactured by Cyagen Biosciences Inc. (Guangzhou, China). Trypsin and PBS were obtained from Sigma Aldrich Inc. (St. Louis, MO, USA).

### 3.2. Preparation of Porous Zirconia Ceramic

The starting materials, 3Y-TZP and stearic acid at 0 wt %, 5 wt %, and 10 wt %, were mixed together by alcohol-based ball milling for 12 h, respectively. The mixtures were pressed at a pressure of 4 MPa, followed by a cold isostatic pressing at 200 MPa. Furthermore, the samples were heated up to 800 °C at a rate of 100 °C/h and kept for 1 h to decompose the stearic acid completely and then sintered at 1450 °C for 2 h.

### 3.3. Characterization

X-ray diffraction spectroscopy (Rigaku, D/MAX-2550V, Tokyo, Japan) was employed to analyze the phase composition. The polished surface and fracture surface morphologies were examined by scanning election microscopy (Hitachi, S-2500N, Tokyo, Japan). Mean pore size distributions were analyzed using a mercury porosimeter (Micromeritics Autopore IV 9500, Atlanta, Georgia, GA, USA). A Vernier caliper was used to determine the linear shrinkage by measuring the diameter of samples before and after sintering. The linear shrinkage was calculated by:

(2) $$(L_{0}-L_{1})/L_{0}\times 100\%$$

where $L_{0}$ is the diameter of samples before sintering and $L_{1}$ is the diameter of samples after sintering. The relative density was measured by the Archimedes method. The surface toughness was measured with the surface profile meter (Surfcom 130A, Tokyo, Japan). A nano-indentation tester (MTS, Palo Alto, California, CA, USA) was applied to analyze the Vickers hardness and elastic modulus. The hardness was calculated by the equation:

*H_v_* = 1.8544*P/d*^2^ (3)

where *H_V_* is the Vickers hardness, *P* is the load and *d* is the diagonal of the indentation.

The elastic modulus is inferred by using the equation:

*E* = 0.45/(*a*/*b* − *a*/*b*_1_) (4)

where *E* is the elastic modulus, b is the length of the shorter diagonal, *b*_1_ is the length of the longer diagonal and a is the length of the crack.

One-way analysis of variance (ANOVA) and Tukey’s multiple comparison test were used to analyze the data. The significance was set at 0.05.

### 3.4. Attachment and Proliferation of rBMSCs

rBMSCs were seeded onto the as-prepared zirconia ceramic in 12-well plates with a density of 2 × 104/well, except the CCK-8 experimental group (5 × 103/well) supplemented with 1 mL DMEM in every well. At time points on day one and day five, rBMSCs were washed with PBS gently and fixed in 4% glutaraldehyd for 2 h. After dehydration in graded ethanol of 30%, 50%, 70%, 80%, 90% and 100% concentrations, rBMSCs were dried in air for 8 h and sputtered with gold prior to observation.

The proliferation of rBMSCs was assessed using a Cell Counting Kit 8 (CCK-8). Cells were seeded in 12-well plates with a density of 5 × 103/well and incubated in fresh DMEM for one, three, and five days. Then rBMSCs were cultured for another 4 h in CCK-8 solution (100 μL WST) in a fully humidified atmosphere with 5% CO_2_ at 37 °C. The absorbance of the supernatant was measured by an ELISAS at a wavelength of 450 nm.

## 4. Conclusions

Porous zirconia ceramics have been prepared with the starting materials, 3Y-TZP and stearic acid. The mechanical properties of zirconia ceramics and proliferation of rBMSCs were investigated as a function of porosity. Our results show that the relative density gradually decreases, and the linear shrinkage increases with the stearic acid (pore-forming agent) content. The pore shape and size varies with the stearic acid content, exhibiting a remarkable effect in promoting the attachment and proliferation of rBMSCs. The porous zirconia has superior biocompatibility, especially for the specimen with a 16% porosity. These results reveal that porous zirconia ceramics are potential materials for further bioactive application.

## Figures and Tables

**Table 1:** The linear shrinkage, relative density, and porosity of samples.

Samples	0 wt %	5 wt %	10 wt %
Linear shrinkage [%]	21.6	21.8	22.1
Relative density [%]	98.9	94.2	84
Porosity [%]	1.1	5.8	16
Roughness (*R*_a_) [nm]	20	53.9	70.7
